# *Candida* gut colonization, yeast species distribution, and biofilm production in *Clostridioides difficile* infected patients: a comparison between three populations in two different time periods

**DOI:** 10.1007/s42770-021-00512-4

**Published:** 2021-07-15

**Authors:** Grazia Brunetti, Alessandro Giuliani, Anna Sara Navazio, Camilla Paradisi, Flavia Raponi, Libenzio Adrian Conti, Giammarco Raponi

**Affiliations:** 1 Department of Public Health and Infectious Diseases, Sapienza University of Rome, Rome, Italy; 2 Department of Environment and Health, Istituto Superiore Di Sanità, Rome, Italy; 3 Department of Molecular Medicine, Laboratory of Microbiology and Pasteur Institute-Cenci Bolognetti Foundation, Sapienza University of Rome, Rome, Italy; 4 Confocal Microscopy Core Facility, IRCSS Bambino Gesù Pediatric Hospital, Research Center, Rome, Italy

**Keywords:** *Candida*, *Clostridioides difficile*, *Candida albicans*, Biofilm, Seasonality

## Abstract

*Candida* gut colonization and yeast biofilm production capacity were investigated, by means of XTT reduction assay, in *Clostridioides difficile* infected (CDI) patients, in non-CDI diarrheic patients, and in healthy donors in two different time periods (2013–2015 and 2018–2019 respectively). *Candida* gut colonization was significantly (*p* < 0.001) associated to *C. difficile* infection, and to patients infected with hypervirulent *C. difficile* strains bearing the *tcdC* deletion at nucleotide 117 (*p* = 0.0003). Although there was not a prevalent yeast species in CDI patients, *C. albicans* was the species significantly (*p* < 0.001) associated to both the infections sustained by the non-hypervirulent *C*. *difficile* strains and those caused by the hypervirulent strain (*p* = 0.001). The biofilm production by the yeasts isolated from the CDI patients and from non-CDI diarrheic patients did not differ significantly. However, a significantly (*p* = 0.007) higher biofilm production was observed in the *Candida* strains, particularly *C. albicans*, isolated from healthy donors compared to that of the yeasts cultured from CDI patients. Seasonal occurrence was observed in the isolation rate of CDI and non-CDI diarrheic cases (*p* = 0.0019), peaking in winter for CDI patients and in spring for non-CDI diarrheic patients. Furthermore, seasonality emerged in the gut colonization by *Candida* of CDI patients in the winter. It seems, therefore, that the reduced capacity of biofilm production by *Candida* strains isolated from CDI patients might have a role in the development of *C. difficile* infection, probably facilitating the spread of the bacteria into the gut thus amplifying their pathogenic action.

## Introduction

*Candida* and *Clostridioides difficile* are two opportunistic pathogens residing in the human gut. *C. difficile* is a Gram-positive, obligate anaerobic, an endospore-forming bacterium that is one of the causes of infectious diarrhea developing in rapid spread, mostly associated with the use of broad-spectrum antibiotics and antacid therapy. The infection frequently occurs in older age hospitalized patients, in residents of long-term care facilities, in subjects suffering from immune-compromisation, malignancies, inflammatory diseases of the gastrointestinal mucosa, and previous *C. difficile* infection (CDI). Concomitantly with the disruption of the normal gut microbioma, *C. difficile* colonizes the large intestine producing the toxins primarily responsible for the associated symptoms [1]. *Candida* spp. and in particular *C. albicans* can be part of the gut microbiome of healthy individuals but the clinical relevance of such evidence is not known, appearing mostly to be commensals and non-pathogenic, unless the host defense system is compromised [2]. Several studies reported an association between CDI and candidemia [3, 4], and recent CDI was one of the factors independently associated with candidemia [5]. The common pathogenetic pathway could be found on the alteration of the human microbiome induced by antimicrobial therapy, which might favor both CDI and persistence of *Candida* in the gastrointestinal (GI) tract [6]. In turn, the extensive mucosal damage caused by the toxins produced by *C. difficile* might allow the dissemination of the yeast in the bloodstream. The virulence of *C. albicans* depends on the growth in yeast, pseudohyphal, and hyphal forms and to the capacity to survive in a biofilm state. The latter could be a phenomenon of paramount importance since the microenvironment of *C. albicans* biofilms can favor the growth of anaerobic microorganism such as *Clostridium perfringens* under normally toxic aerobic conditions [7]. In a previous study, we evidenced a link between CDI and *Candida* colonization of the gut [8]. Nowadays, different authors evidenced either positive or negative correlation between CDI and *Candida*. Since the debate is still open, in this study, we investigated the differences in the *Candida* gut colonization of CDI patients, of non-CDI diarrheic patients, and of healthy donors in two different periods with different epidemiological CDI diffusion. Furthermore, we wanted to investigate whether the metabolically active biofilm production by *Candida* differed among strains isolated from the three study populations.

## Materials and methods

In this control study, 482 stool specimens were sampled at the 1300 beds-teaching hospital Policlinico “Umberto I” in Rome, Italy. The study analyzed existing laboratory data that were anonymized before being included in the study database. Stool specimens were collected at the hospitalization site, placed at 4 °C in the absence of preservative substances, and processed within 30 min from the collection. Diarrheic stool specimens were defined as ≥ 5 of the Bristol stool chart [9], considering a rejection criterion if the score was < 5, except in the case of specimen collection from healthy donors. In order to compare homogenous populations, 243 consecutive diarrheic stool specimens were collected between November 2013 and March 2015, as well as 239 stool specimens, including 106 stool samples from healthy donors and 133 diarrheic stool specimens positive for the presence of toxinogenic *C. difficile*, in the period between May 2018 and May 2019. All the stool samples were investigated for the presence of toxinogenic *C. difficile* following a two steps diagnostic algorithm, in which all the samples displaying positive reaction to the immune-chromatographic GDH assay screening (C. diff quik chek®, TechLab, Blacksburg, USA) were analyzed for the presence of toxin B (*tcdB* gene), binary toxin (*cdtA* gene), and a *tcdC* deletion gene at nucleotide 117 (NAP-BI-027 presumptive positive), by RT-PCR (Xpert® C. difficile, Cepheid, Sunnyvale, USA). Quantification of *Candida* gut colonization of all the fecal samples was achieved by diluting 10 µL of diarrheic stool sample or a net loop of solid stool sample (approx. 10 µg) in 1 ml of sterile 0.9% NaCl solution, plating 10 µL on Sabouraud dextrose agar supplemented with chloramphenicol (Oxoid spa, Milan, Italy). Plates were incubated for at least 24 h and up to 5 days at 30 °C. Yeast growth was defined as positive if ≥ 10^4^ CFU/ml grew from the stool samples [2]. The yeasts were typed by matrix-assisted laser desorption/ionization assay (MALDI-TOF Bruker Daltonik GmbH, Bremen, Germany), using an extraction process by means of ethanol treatment, followed by formic acid and acetonitrile extraction and accepting score values ≥ 1.8 [10, 11]. Yeast strains were frozen and stored at − 80 °C in Microbank™ Storage system (Pro-Labs Diagnostics, Richmond Hill, Ontario, Canada) until analysis. The formation of metabolic active fungal biofilm of Candida strains was measured in vitro by means of 2,3-bis (2-methoxy-4-nitro-5-sulfophenyl)-2H-tetrazolium-5-carboxanilide (XTT, Sigma-Aldrich s.r.l., Milan, Italy) reduction assay with slight modifications [12]. Briefly, each *Candida* strain in the log-phase of growth (200 μl of 10^6^ cells/ml in RPMI 1640) was plated on flat-bottomed 96-well polystyrene microtiter plates and incubated at 37 °C for 48 h. After incubation, the plates were washed with sterile PBS and added with 100 µL of a solution 0.67 g/L XTT-1 µM menadione (Sigma-Aldrich). The resulting color change was spectrophotometrically quantified at 490 nm with a microtiter plate reader (Eti System Reader, Bio-Tek Instruments Inc., Winooski, VT, USA). To standardize the method, the results were expressed in terms of Biofilm Index (BI) [13] calculated as the ratio between the absorbance test strain and the absorbance of a stable biofilm-producing *Candida albicans* strain (*C. albicans* SA40, kind gift from dr. F. De Bernardis, National Health Institute of Rome) [14]. Three different categories of patients, i.e., patients carrying toxinogenic *C. difficile* (CDI), non-CDI diarrheic patients, and healthy donors, were considered in two different time intervals (years 2013–2015 vs. 2018–2019). Informed written patients’ consent was not required because of the observational nature of the study. The Ethical Committee of “Sapienza” University of Rome (Prot. 13/18) approved the study.

### Statistical analysis

The production of biofilm by the yeast isolates was expressed as the mean ± SD of the BI value. Statistical analyses were performed using the SAS/STAT software. We adopted a chi-square correlation metrics when in presence of categorical variables; thus, inferential analysis was based on *χ*^2^ test or Fisher’s exact test when needed. Inferential statistics on continuous variables was performed by means of Student’s *t* test for two group comparisons and by analysis of variance (ANOVA) when in presence of more than two groups. An *α* error < 0.05 was accepted as significance threshold.

## Results

### *Clostridioides difficile* in diarrheic patients

In this case–control study, 482 stool specimens were collected. On basis of year isolation (2013–2015 vs. 2018–2019), these samples were analyzed and compared. The statistical analysis confirmed that the two studied temporal phases were homogeneous by the relative percentage of the number and age of patients’ distribution, the month of isolation, and the number of *Candida* strains tested for biofilm production capacity. In the years 2013–2015, diarrheic stool specimens (nr = 243) were investigated for the positivity to *C. difficile.* Among these samples, 136 derived from non-CDI diarrheic patients (median age 61 ± 22.41, 44.12% female), while 107 resulted positive to the RT-PCR test. Of these,70 patients suffered from non-hypervirulent CDI (median age 70 ± 18.01, 60% female), and 34.58% displayed the *tcdC* deletion at nucleotide 117 (NAP-BI-027 presumptive positive, median age 56.76 ± 15, 56.76% female). In 2018–2019, 239 stool specimens were analyzed: 133 samples tested positive to RT-PCR, 105 resulted positive to non-hypervirulent *C. difficile* (median age 71 ± 17, 49.1% female) with 21% positivity for hypervirulent *C. difficile* (median age 75.8 ± 18.01, 60% female), and 106 samples were derived from healthy donors (median age 50 ± 20, 50% female). An epidemiological shift of the toxinogenic profile of CDI diffusion was observed among the two periods. Indeed, a statistically significant increase in the prevalence of the non-hypervirulent strains (i.e., strains not bearing the *tcdC* gene deletion at nucleotide 117) was observed in 2018–2019 compared to 2013–2015 (chi-square = 5.49, *p* = 0.01). Moreover, a higher incidence of infections sustained by *C. difficile* was confirmed in female (chi-square = 5.21, *p* = 0.02) and elderly patients (median age = 71.40; chi-square 25.74, *p* < 0.001). The age was significantly higher in hypervirulent *C. difficile* than non-hypervirulent *C. difficile* patients (74.76 vs. 65.91, respectively) (*p* < 0.001).

### *Clostridioides difficile *and *Candida* colonization

**Fig. 1:**
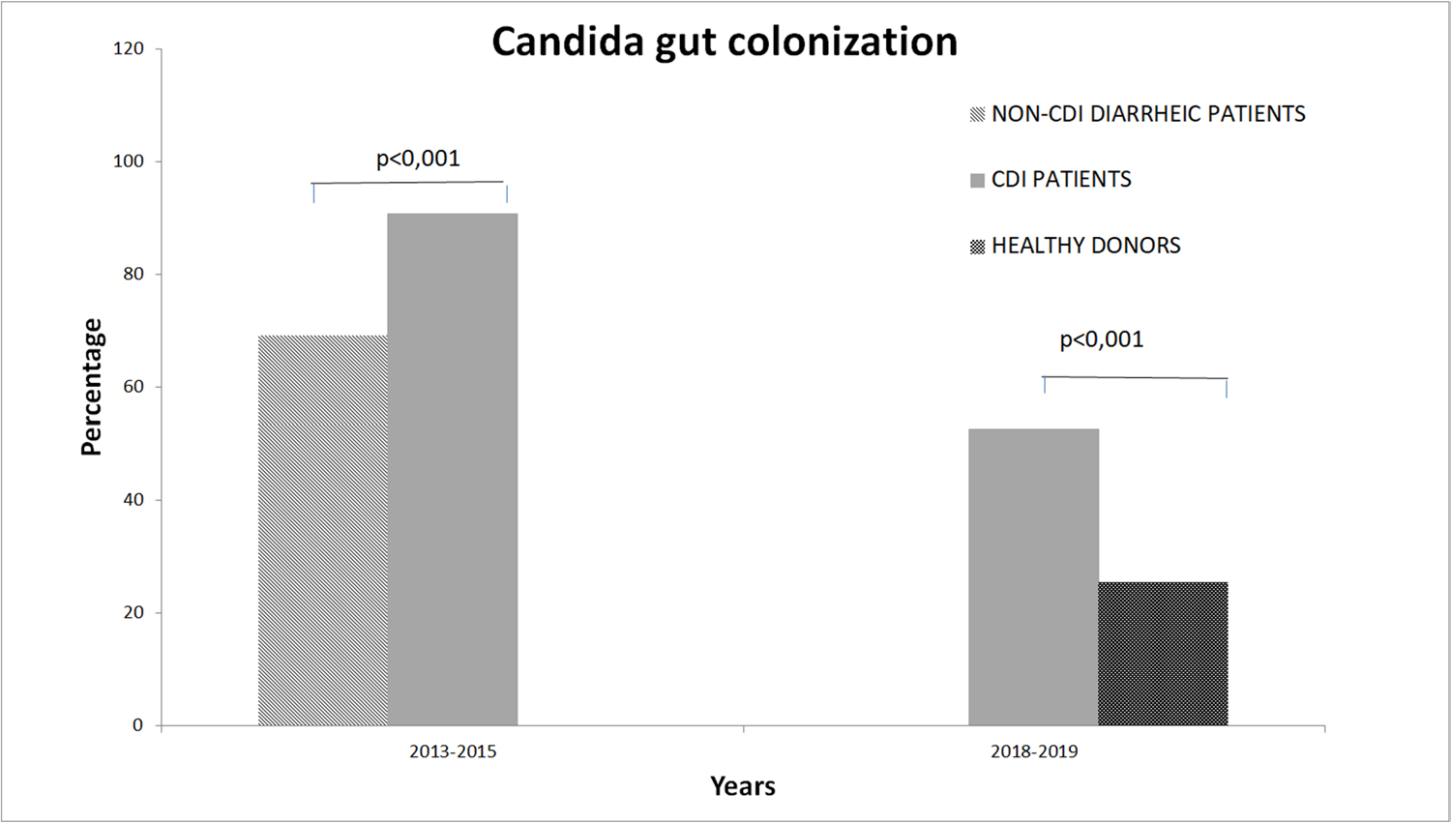
Association between *Candida* colonization and CDI infections. The statistical association emerged when comparing the percentages of *Candida* gut colonization in CDI patients and in non-CDI diarrheic patients (chi-square = 16.51, *p* < 0.001), as well as when comparing the percentages of colonization in CDI patients and in healthy donors (chi-square = 18.04, *p* < 0.001)

*Candida* colonization of the gut was investigated in all 482 patients and found in 288 samples. Data analysis showed that *Candida* colonization varied in the different group of patients. In fact, while *Candida* colonization rate was of 25.47% in the healthy donors group (nr = 106), the general rate observed in all diarrheic stool samples, collected from both CDI patients and non-CDI diarrheic patients, approached 69% in both groups. Considering the data in the different phases of the study (2013–2015 vs. 2018–2019), it emerged that in the first period, *Candida* colonized the intestinal gut of 90.65% of CDI patients (nr = 107) and of 69.12% of non-CDI diarrheic patients (nr = 136). This relative frequency difference was statistically significant (chi-square = 16.51, *p* < 0.001) indicating a strong specific association between *Candida* colonization and CDI. Furthermore, in the second period when comparing *Candida* gut colonization of CDI patients and of healthy donors, this association was statistically confirmed (chi-square = 18.04, *p* < 0.001) (Fig. 1). In fact, while in healthy donors, *Candida* colonized only 25.47% of 106 enrolled patients, in CDI patients, *Candida* gut colonization was observed in 52.63% of the 133 samples. Analyzing the whole set of patients and comparing the three different categories of patients, *Candida* gut colonization was confirmed significantly associated to CDI (chi-square = 66.4, *p* < 0.0001). Furthermore, this association was also confirmed when studying only the subgroup of patients infected with *C. difficile* strains bearing the *tcdC* gene deletion at nucleotide 117 (chi-square = 12.81, *p* = 0.0003). Stratifying the data according to the species of colonizing *Candida* in the overall analysis, *C. albicans* was the most prevalent species (58.7%), followed by *C. glabrata*, both in CDI patients and in non-CDI diarrheic patients (52.1% and 22%, respectively). A similar situation was observed in the group of healthy donors in which *C. albicans* (59.2%) was the species most frequently isolated followed by *C. glabrata* (18.5%). The distribution of *Candida* species in the different periods and in the different groups was homogeneous, witnessing that there was no species prevalence in the different groups. However, when the statistical analysis included all the enrolled patients, it emerged that *Candida albicans* was the species most frequently associated with CDI both in the analysis between CDI and non-CDI diarrheic patients (chi-square = 22.12, *p* = 0.0005) and between CDI patients and healthy donors (chi-square = 20.9177, *p* = 0.0008) and in the overall analysis (chi-square = 80.86, *p* < 0.001) (Tables 1 and 2). This association was also reflected in the correlation between *C. albicans* colonization in patients infected by the *C. difficile* strains bearing the *tcdC* gene deletion at nucleotide 117 (hypervirulent) (chi-square = 20.47, *p* = 0.001).

**Table 1:** Gut colonization by *Candida* species. In the overall analysis, *Candida albicans* was the species most frequently associated with CDI (chi-square = 80.86, *p* < 0.001). *Other species included *C. guilliermondii*, *C. krusei*, *C. sake*, and *C. lusitaniae*. In parenthesis are reported the number of cases

Total analysis			
	CDI patients	Non-CDI diarrheic patients	Healthy donors
*Candida albicans*	40.83% (98)	36.03% (49)	15.09% (16)
*Candida glabrata*	15.42% (37)	11.03% (15)	4.72% (5)
*Candida parapsilosis*	1.25% (3)	2.94% (4)	1.89% (2)
*Candida tropicalis*	4.58% (11)	2.21% (3)	0.94% (1)
Other*	7.50% (18)	16.91% (23)	2.83% (3)
No *Candida* colonization	30.42% (73)	30.88% (42)	74.53% (79)

**Table 2:** Gut colonization by *Candida* species among CDI and non-CDI diarrheic patients in the period 2013–2015 and between CDI patients and healthy donors in the period 2018–2019. *Candida albicans* was the species most frequently associated with CDI in the two different periods (chi-square = 22.12, *p* = 0.0005 and chi-square = 20.9177, *p* = 0.0008 respectively). *Other species included *C. guilliermondii*, *C. krusei*, *C. sake*, and *C. lusitaniae.* In parenthesis are reported the number of cases

	2013–2015		2018–2019	
	CDI patients	Non-CDI diarrheic patients	CDI patients	Healthy donors
*Candida albicans*	56.07% (60)	36.03% (49)	28.57% (38)	15.09% (16)
*Candida glabrata*	17.76% (19)	11.03% (15)	13.53% (18)	4.72% (5)
*Candida parapsilosis*	0.93% (1)	2.94% (4)	1.50% (2)	1.89% (2)
*Candida tropicalis*	2.80% (3)	2.21% (3)	6.02% (8)	0.94% (1)
Other*	13.08% (14)	16.91% (23)	3.01% (4)	2.83% (3)
No *Candida* colonization	9.35% (10)	30.88% (42)	47.37% (63)	74.53% (79)

### Seasonality

**Fig. 2:**
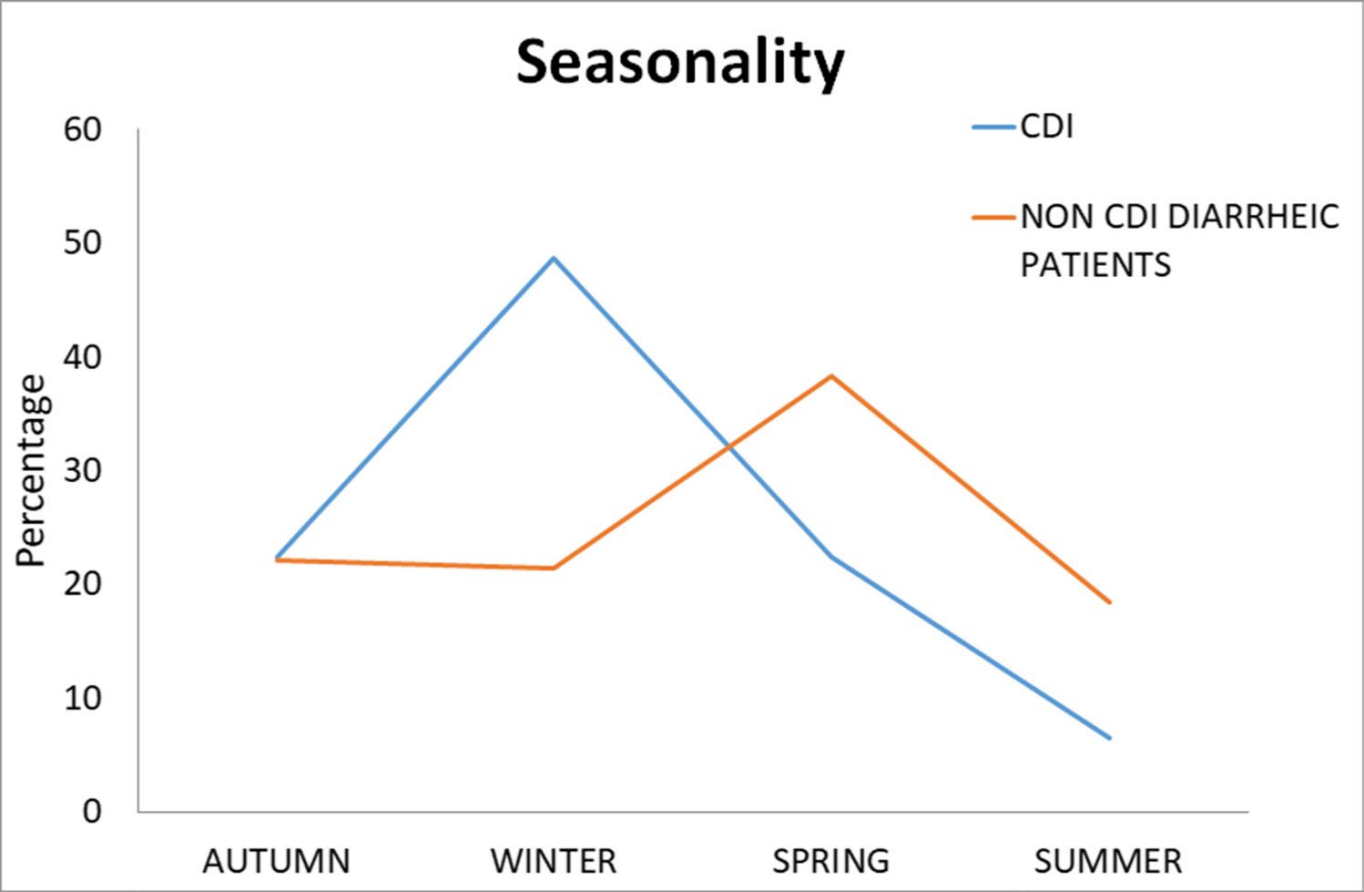
Isolation rate of CDI and non-CDI diarrheic cases. Statistical association emerged in the distribution of the percentage of case (chi-square = 14.86, *p* = 0.0019). Seasonality data were referred to four-season periods as follows: winter, January 1 until March 31; spring, April 1 until June 30; summer, July 1 until September 30; autumn, October 1 until December 31

Differential distribution of cases among CDI patients and non-CDI diarrheic patients was observed stratifying data according to the months of isolation (chi-square = 14.86, *p* = 0.0019 relative to non-CDI-diarrheic patients). In particular, in the first period of study, the highest CDI isolation rate was observed during the winter, while non-CDI diarrheic cases peaked in spring and the relative distribution of cases was specular. While during autumn, the percentage of episodes was relatively uniform, in the summer, the number of events involving *C. difficile* was lower (Fig. 2). Moreover, *Candida* gut colonization in CDI patients evidenced seasonality as well. In fact, despite the percentage of *Candida* colonization remained steady in the various seasons, the analysis of the relative yes/no distribution showed that the highest percentage of *Candida* colonization reached 88.68% in winter and 82.00% in spring (chi-square = 22.22, *p* < 0.0001) (Table 3).

**Table 3:** Global analysis of the yes/no relative distribution of *Candida* gut colonization in CDI patients. Seasonality data were referred to four-season periods as follows: winter, January 1 until March 31; spring, April 1 until June 30; summer, July 1 until September 30; autumn, October 1 until December 31

Season	*Candida* gut colonization in CDI patients (%, number of cases)	
	No	Yes
Autumn	40.30% (27)	59.70% (40)
Winter	11.32% (6)	88.68% (47)
Spring	18.00% (9)	82.00% (41)
Summer	44.29% (31)	55.71% (39)

### Biofilm production

**Fig. 3:**
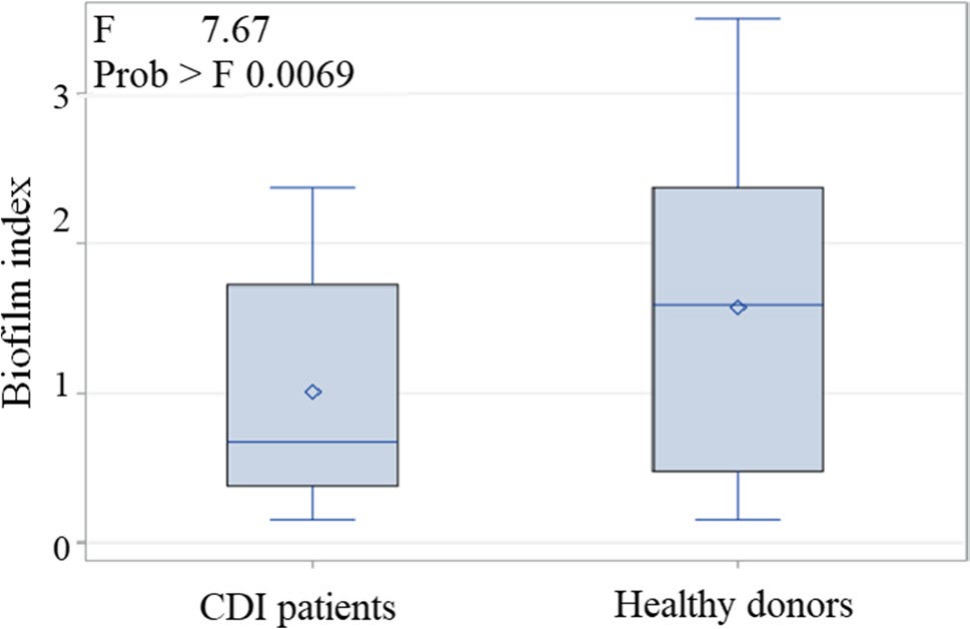
Comparison of biofilm production capacity of *Candida* (expressed in terms of Biofilm Index) between CDI patients and healthy donors. The statistical analysis showed a higher biofilm production by *Candida* strains isolated from healthy donors compared to CDI patients (*p* = 0.007)

The biofilm production was performed on 234/288 *Candida* strains that were viable after thawing (140 isolated from the stools of CDI positive patients, 67 *Candida* strains isolated from non-CDI diarrheic patients, and 27 *Candida* strains isolated from healthy donors) through XTT reduction assay, equating the percentage of the *Candida* species distribution. Results were expressed in terms of Biofilm Index (BI). From the comparison between the three studied patients’ categories, no statistically significant differences emerged in the production of BI, as well as in the comparison between CDI patients and non-CDI diarrheic patients. However, the statistical analysis showed a higher biofilm production by *Candida* strains isolated from healthy donors compared to CDI patients (*p* = 0.007) (Fig. 3). *C. albicans* had the highest capacity in producing biofilm compared to the other *Candida* species. However, there was no higher biofilm production by *C. albicans* isolated from CDI patients compared to that of non-CDI diarrheic patients. Instead, *C. albicans *biofilm production was significantly enhanced when the strains were isolated from healthy donors compared to CDI patients (*p* = 0.0006). This increase was not observed when biofilm production capacity was measured in strains of *Candida albicans* isolated from healthy donors compared to the biofilm production in non-CDI diarrheic patients.

## Discussion

It is widely accepted that antibiotic-induced alteration of the intestinal flora might have a positive effect on *Candida* spp. gut colonization. Several studies have focused on the prevalence of *C. albicans* in *C. difficile* infected patients, yet the nature of the interactions between these microorganisms is still controversial [15, 16]. In a previous study, we observed that CDI-positive patients had high rates of *C. albicans* gut colonization [8], and another paper reported non-albicans *Candida* (NAC) colonization in a trial on 548 patients with CDI [17]. Conversely, Manian et al. reported a negative association between *Candida* spp. overgrowth and CDI [16]. In our study, despite similar patient characteristics in two different periods, the fecal samples from patients with CDI demonstrated a consistent and significant *Candida* gut colonization that was not present among non-CDI diarrheic patients. Moreover, we found strong correlation between CDI and *Candida *spp. colonization due to the observation that the stool samples of healthy donors displayed a significantly lower incidence of *Candida* colonization. In particular, if we extended the study to the whole group of enrolled patients, *Candida albicans* was the yeast most frequently isolated in CDI patients. Nowadays, this yeast is considered a common gut commensal of humans, influencing the composition of the bacterial microbioma [18]. However, in our study, only 15.09% of the healthy donors had *C. albicans* colonization, though it was the species most frequently isolated (59.2%). Therefore, we can speculate that the higher and statistically significant intestinal colonization by *Candida* spp. in CDI patients, and in particular by *C. albicans*, might be promoted by the antibiotic therapy. As widely described in the literature, the antibiotics can be considered the principal risk factor for CDI with non-specific effect on gut microbioma [19–21]. Several studies found seasonality of CDI with different peaks depending on the different geographical area [22]. Seasonal climate [23] and the correlation with other respiratory infections have been invoked to explain such findings [24, 25]. Suda et al. evidenced a higher prescription rate of antibiotics during winter [26], supporting the hypothesis that seasonal antibiotic prescriptions rate might have contributed to the seasonality of CDI observed in our study, a finding that coincided with the highest percentage of *Candida* gut colonization. In the interplay between *C. difficile* and *C. albicans*, the yeast might promote the development of CDI expanding the ecological niches of *C. difficile* so that its vegetative cells might be able to survive to an oxygenated environment. This hypothesis was underscored by the findings of Pim et al. who demonstrated that *C. difficile* can survive to an oxygenated environment when co-cultured with *C. albicans* [15]. The interactions between *C. difficile* and *C. albicans* could be multiple, but the capacity of the yeast to produce biofilm might be of paramount importance in the pathogenic processes of CDI leading to invasion of the mucosal cells [27]. Hence, the ability of *Candida* to produce biofilm could mediate CD adhesion to the gut, thus allowing the release of *C. difficile* toxins in the intestinal mucosa, the dissemination, and the onset of the disease. Therefore, in our study, to ascertain whether CDI was associated to *Candida* strains with higher biofilm-producing capacity, the biofilm production was measured in *Candida* strains isolated from the stools of CDI patients, of non-CDI diarrheic patients, and of healthy donors. No significant differences emerged in the *Candida *biofilm production between *Candida* strains isolated from CDI patients and the strains isolated from non-CDI diarrheic patients. However, a higher biofilm production by Candida strains, in particular C. albicans, was observed when the strains were cultured from healthy donors compared to that of yeasts isolated from CDI patients. It is possible that the intense host inflammatory response caused by toxin hyper production [28] and the intestinal mucosal barrier destruction [29, 30] could inhibit *Candida* biofilm production, thus allowing penetration of CD into the mucosa and manifesting its pathogenic action. Pim et al. demonstrated that, although *C. albicans* allowed *C. difficile* to survive in aerobic ambient, when this organism was co-cultured with *C. albicans*, it produced chemical signals such as p-cresol with inhibitory activity against the *C. albicans* biofilm formation [15]. These latter findings seem to further explain the higher incidence of *C. albicans* isolation in CDI patients observed in our study, together with the observation that biofilm production was not an intrinsic capacity of the Candida strain involved in the infection, rather by the greater frequency of isolation of *Candida* spp., in particular *C. albicans*, in CDI infection. Not in the end, in our study, we observed a change of the toxinogenic profile of CDI diffusion, with a significant increase in the prevalence of the non-hypervirulent *C. difficile* strains (i.e., strains not expressing the *tcdC* gene deletion at nucleotide 117) in the second period of the study compared to the first one. This phenomenon could be explained by the implementation of control and containment measures of this infection applied in our hospital, and it will be the topic of forthcoming studies. Our study suffers of two major limitations. Firstly, being a retrospective analysis, there is a lack of data regarding patients’ clinical features and the antimicrobial treatment received by the single patient before the development of CDI that may have influenced the *Candida* gut colonization. Secondly, the study lacks data regarding the clonality of both *C. difficile* and *Candida* isolates in order to correlate the interactions between the two species.

## Conclusion

*Candida* gut colonization accompanied symptomatic CDI, in particular those sustained by *C. difficile* strains bearing the *tcdC* deletion gene at nucleotide 117. The antibiotic prescription rate could play a key role in the alteration of the intestinal flora. In the winter months, we recorded a greater percentage of *Candida* gut colonization, and, surprisingly, just in these months, we registered the peak of *C. difficile* infections determining the seasonality. In CDI patients, the percentage of *Candida* colonization was three times higher than the percentage of *Candida* colonization in the healthy donors, such as the *C. albicans* colonization. No single isolated species of *Candida* was prevalent in CDI patients, but when considering all the patients enrolled, the greater frequency of isolation of *C. albicans* was associated both to the non-hypervirulent CDI strains and to strains bearing the *tcdC* gene deletion at nucleotide 117. No statistically significant differences emerged in the biofilm production from the comparison of *Candida* strains isolated in healthy donors and diarrheic non-CDI patients, with the notable exception of *C. albicans* that showed an increased biofilm production when isolated from healthy donors compared to CDI patients. The interactions between *C. difficile* and *C. albicans* could be multiple and the infection could progress through various steps allowing the penetration of CD into the mucosa and manifesting the pathogenic action.

## Data Availability

All relevant data are within the paper.
